# Reviewers Acknowledgement

**DOI:** 10.1002/1878-0261.13165

**Published:** 2022-01-05

The Editors of *Molecular Oncology* would like to thank all referees who have given their time and expertise to review articles submitted for publication in Volume 15. *Molecular Oncology* heavily relies on reviewers' careful assessment, constructive criticism and timely response to ensure both quality and fast turnaround times.

The names of these reviewers are listed below; we apologize if we have inadvertently omitted anyone.

Abbosh Chris

Adusumilli Prasad

Agoulnik Alexander I

Ahmad Imran

Ahmed Wareed

Ahmed Zeeshan

Ahn Jinwoo

Alaniz Laura Daniela

Albi Elisabetta

Alexandraki Krystallenia

Alghamdi Mohammed

Alig Stefan

Almeida Raquel

Altieri Barbara

Alus Xiaoli

Alver Tine

Alves Joao

Amat Ramon

Ambros Peter F

Amler Evzen

Andersen Claus

Anelli Luisa

Angelastro James Michael

Ansari Daniel

Apetrei Cristian

Apps John

Aran Veronica

Arbiser Jack

Arcangeli Annarosa

Ariey‐Bonnet Jérémy

Arnaud Philippe

Asangani Irfan

Ashktorab Hassan

Aspenström Pontus

Astolfi Annalisa

Ateeq Bushra

Avanzo Michele

Avery V

Ayuda‐Durán Pilar

Azad Arun

Azencott Chloé‐Agathe

Baba Hideo

Baccarini Manuela

Baniahmad Aria

Banys‐Paluchowski Maggie

Barchi Marco

Barderas Rodrigo

Bartel Karin

Barthet Valentin JA

Bartkova Jirina

Bartolomé Ruben A

Bartsch Joerg Walter

Baumann Michael

Baumgartner Martin

Beane Freemann Laura

Beggs Andrew

Behrens Jürgen

Ben‐David Yaacov

Berasain Carmen

Berg Kaja CG

Bergmann Michael

Berner Kai

Berns Anton JM

Bertolo Riccardo

Best Myron

Best Sarah

Betge Johannes

Biermann Jana

Bin Yu

Birch Joanna L

Bishai William R

Blandino Giovanni

Boegel Sebastian

Bol Guus M

Bon Giulia

Boncela Joanna

Bonci Desiree

Borggrefe Tilman

Borkowska Edyta

Bose Chinmoy

Boyano María Dolores

Boye Kjetil

Braithwaite Antony

Brekken Rolf

Bröer Stefan

Brumatti Gabriela

Brunetti Lorenzo

Brustugun Odd Terje

Buchhalter Ivo

Buldini Barbara

Burnstein Kerry L

Butler Lisa M

Caballero Otavia

Cabel Luc

Cai Changmeng

Cai Gengming

Cai Shenglan

Calin George A

Callari Maurizio

Camero Simona

Campanella Michelangello

Campbell Kristeen

Campello Silvia

Canale Matteo

Cao Minghui

Capella Gabriel

Cappelletti Vera

Caraglia Michele

Cardaci Simone

Carneiro Fatima

Carraway Kermit L

Carreras‐Puigvert Jordi

Carroll Jason S

Casal J Ignacio

Casola Stefano

Celetti Angela

Cervantes Andres

Chada Kiran

Chakraborty Anirban

Chakraborty Goutam

Chakravorty Soumen

Challagundla Kishore Babu

Chalmers Anthony J

Chang Suhwan

Chanhee Kang

Chapman Mike

Charan Manish

Chatterji Urmi

Chauhan Sudhir Kumar

Chemi Francesca

Chen BaoQing

Chen Hui

Chen Li‐Han

Chen Pai‐Sheng

Chen Wenlin

Chen Xi

Cheng Shiyuan

Cheng Zhen

Chieffi Paolo

Choi Woonyoung

Choi Yu Suk

Chou Chih‐Hung

Choudhuri Tathagata

Choyke Peter

Christensen Emil

Christian Sherri

Christina Voelkel‐Johnson

Christopher Jana

Chu Simon

Chuma Makoto

Ciasca Gabriele

Ciniselli Chiara

Ciribilli Yari

Clague Michael

Clarke Robert

Classens F

Clifford Gary M

Coccaro Nicoletta

Cohen David S

Coleman Michel

Collis Spencer

Comoglio Paolo N

Cong Yu‐Sheng

Cortes Felipe

Corujo David

Costa Valerio

Costanzo Vincenzo

Coulie Pierre

Crea Francesco

Cronauer Marcus Victor

Culig Zoran

D'Agostino Vito

Dambrosio Lorenzo

Dang Ningxin

D'Angiolella Vincenzo

Das Chandrima

Dasgupta Subhamoy

Daver N

David Leonor

Davidson Ben

De Bortoli Michele

De Maglio Giovanna

De Maria Ruggero

De Nardo Dominic

De Petris Luigi

Debnath Jayanta

Dejardin Emanuel

Deng Qu

Dequiedt Franck

Deshpande Ravindra

Di Fazio Pietro

Di Nicola Massimo

Di Stefano Giuseppina

Diamond Jennifer

Dika Emi

Dikalov Sergey I

Dillon Richard

Dimas Konstantinos

Dimopoulos Konstantinos

D'Incalci Maurizio

Ding Wen‐Xing

Ding Xiangya

Djouder Nabil

Dolci Susanna

Done Susan J

Dopeso Higinio

Dowlati Afshin

Drago Joshua

Dragomir Mihnea P

Driescher Caroline

Duarte Henrique O

Duensing Stefan

Duran Raul V

Durocher Francine

Dykhuizen Emily

Ebi Hiromichi

Ebinger Martin

Edlich Frank

Efeyan Alejo

Egger Gerda

Ehata Shogo

Eikesdal Hans Petter

Eiring Anna

Eisenstat David

Ekert Paul

Engebraaten Olav

Enguita Francisco

Enroth Stefan

Enserink Jorrit

Espina Carolina

Esteller Manel

Eyal Eran

Eyers Patrick

Fabbri Muller

Fabbris Linda

Faller William James

Fanciulli Maurizio

Fang Lan

Fatemeh Derakhshan

Feltham Rebecca

Fernandez‐Capetillo Oscar

Fernández‐Zapico Martín

Ferretti Elisabetta

Filipska Martyna

Finetti Francesca

Flanagan James M

Fleischer Thomas

Fleischhacker Michael

Florio Tullio

Fon tacer Klementina

Font de Mora Jaime

Forshner Andrea

Fosdahl Anne Marthe

Fouassier Laura

Foukakis Theodoros

Fraga Mario Fernández

Francavilla Chiara

Frank Malene

Fraser Michael

Friedman E

Frigola Joan

Froeling Fieke

Fucà Giovanni

Fujiwara Noriko

Funakoshi‐Tago Megumi

Fung Ka Yee

Funk Maja

Gabrielli Brian

Gagliano Teresa

Galindo Rene

Gambus Agnieszka

Gammoh Noor

García de Herreros Antonio

Garinet Simon

Garkavtsev Igor

Garrison Louis

Gautreau Alexis

Gerada Chelsea

Ghayad Sandra E

Giavazzi Raffaella

Giovannetti E

Gires Olivier

Godbout Roseline

Goh Boon‐Cher

Gomes Catarina

Gomez Miragaya Jorge

Gomez‐Roman Natividad

Gonçalves Emanuel

Gonzalez Wildredo

Gonzalez‐Martin Alice

Gopalakrishnan Dharmesh

Gorgoulis Vassilis

Gorzelanny Christian

Grani Giorgio

Gregory Philip

Grignani G

Grigoriadis Anita

Griñan Lisón Carmen

Groom Joanna R

Gross Julia

Gu Yifeng

Guan Jikui

Guillermet‐Guibert Julie

Gullberg Donald

Guo Tiannan

Guren Tormod

Gussgard Syljuåsen Randi

Gutschner Tony

Ha Geun‐Hyoung

Haakensen Vilde Drageset

Haass Nikolas K

Hagen Thilo

Halbrook Chris

Hamerlik Petra

Hanash SM

Hansen Torben F

Hartkopf Andreas

Hasnain Sumaira

Hausch Felix

Hayami Shinya

Hayashi Yujiro

Hayes Daniel F

Hedenfalk Ingrid

Hedman HÃ¥kan

Hegedus Balazs

Helland Aslaug

Hellström Mats

Henrique Rui

Herbert Katharine

Hess Jochen

Hillmer Axel

Hjorth Lars

Hofman Paul

Hogg Simon

Hong Andrew

Hong Chibo

Hoon Dave SB

Hopper John L

Hoppe‐Seyler Felix

Hsu Ping‐Chih

Hsu Tsung‐I

Hu Baoli

Hu Jiancheng

Hu Ying

Huang Eric

Huang Jian

HuangYang Meng

Hühn Daniela

Hunter Jill

Hunter Tony

Hurley Paula

Ichim Gabriel

Ignatius Myron

Ilter Didem

Inturi Raviteja

Iorio Francesco

Iqbal Mohammad Askandar

Irwin Jane A

Isaacs John

Ishimoto Takatsugu

Italiano Antoine

Jaakkola Panu

Jackstadt Rene‐Filip

Jaffe Elaine S

Jaffrey Samie

Jansen Maurice

Jardim Denis L

Javierre Biola

Jeronimo Carmen

Jin Victor X

Joensuu Heikki

Johnsen Steven

Juhlin Carl Christofer

Jungwirt Ute

Kagohara Luciane T

Kandasamy Palanivel

Kantelhardt Eva Johanna

Karn Thomas

Karteris Emmanouil

Kassiotis George

Katayama Ryohei

Kaulich Manuel

Kaushik Vivek

Kawakami Hisato

Kazi Julhash U

Kearney Conor

Keller Laura

Kenneth Niall S

Kersten Kelly

Kietzmann Thomas

Kim Cheorl‐Ho

Kim Ja‐Eun

Kim Yesol

Kim Yonghyun

Klinakis Apostolos

Klinge Carolyn M

Knutsen Erik

Kodous Ahmad

Koehne de Gonzalez Anne

Koinuma Daizo

Kong Lu

Koopman Bart

Koppers‐Lalic Danijela

Korkaya Hasan

Kostareli Efterpi

Kotula Leszek

Kreuter Alexander

Krueger Achim

Kruyt Frank AE

Ksiazek Krzysztof

Kuenkele Annette

Kulasinghe Arutha

Kumari Sonam

Künstner Axel

Kuster Bernard

Kuznetsov Vladimir A

Kwok Hoi‐Hin

Kyprianou Natasha

Laia Caja

Lal Girdhari

Lalaoui Najoua

Lalloue Fabrice

Lam Henry

Lamy Pierre‐Jean

Lancaster Graeme

Landberg Goran

Lange Tobias

Larue Lionel

Laurent‐Puig Pierre

Lawlor Rita T

Leandersson Karin

Lecona Emilio

Lee Erinna

Leeansyah Edwin

Lena Anna Maria

Lenos Kristiaan

Leszczynska Katarzyna

Lev Sima

Levis Mark

Lewis Robert

Li Qing

Liao Chengcheng

Lim Bora

Lin Jung‐Chun

Linardic Corinne M

Lindbjerg Andersen Claus

Lindner John

Liou Geou‐Yarh

Liou Yih‐Cherng

Liu Zhonghua

Lobo João

Lopez‐Contreras Andres Joaquin

Lord Chris

Lu Haojie

Lu Yong‐Jie

Lueckerath Katharina

Luo Jun

Luo Yanwei

Luo Zhijun

Macurek Libor

Madeddu Roberto

Mælandsmo Gunhild Mari

Magnani Diogo

Maiuri Maria Chiara

Manapov Farkhad

Mancini Mara

Mantovani Giovanna

Mara Mancini

Maran Avudaiappan

Marangoni Elisabetta

Mardinoglu A

Marie Suely

Marin Oskar

Marin‐Bejar Oskar

Marra Antonio

Martelli Alberto

Martinez Tirado Oscar

Martinez‐Pastor Barbara

Martins L Miguel

Marx Christian

Masuko Takashi

Mayor‐Ruiz Cristina

Mazzilli Sarah

McEwan David

McGrail D

McGuckin Michael

Mcstay Gavin

Medeiros R

Mego Michael

Melino Gerry

Mendes Marta

Michlewski Gracjan

Miękus Katarzyna

Mills Ian

Mineo Marco

Minihane Emma

Miyashita Naoya

Mohar Alejandro

Monk David

Mora Jaume

Morel Etienne

Morgan Gareth J

Morgan Iain

Morris Katherine

Mosquera Orgueira Adrián

Mouliere Florent

Msaouel Pavlos

Muehlenberg Thomas

Mukherji Atish

Müller Jörg

Muller Patricia

Murphy Daniel

Muthana Munitta

Nakai Kenta

Narita Masashi

Nasser Mohd Wasim

Nawaz Muhammad

Nebreda Angel R

Neubauer Hans

Neve Bernadette

Nguyen Paul

Ni Wei

Nicholson Sandra

Niclou Simone P

Nielsen Torsten O

Nishihara Hiroshi

Nishitani Hideo

Niv Masha

Nobili Stefania

Normanno Nicola

Nowak Dawid G

Nsengimana Jeremie

Nylandsted Jesper

Offringa Ite

Oing Christoph

Okamoto Yasuhiro

Oliveira‐Ferrer Leticia

Op de Beeck Ken

O'Reilly Lorraine

Oren Moshe

Ottaiano Alessandro

Owens Philip

Packer Nicolle Hannah

Palacios Jose

Palmer Ruth Helen

Palmieri Dario

Pan Xuan

Pan Yijun

Panatta Emanuele

Pantel Klaus

Pardini Barbara

Pareja Fresia

Parisse Pietro

Park Jong Hoon

Parsons Jason

Pasi Janne

Pateras Ioannis

Peinado Miguel Angel

Perets Ruth

Peschiaroli Angelo

Peterlongo Paolo

Philpott Mike

Piao Hai‐long

Pichler Martin

Pierobon Mariaelena

Pinzani Pamela

Pistoni Mariaelena

Pollock Pamela

Polzer Bernhard

Prat Aleix

Pucci Perla

Pulido Rafael

Qin Fei

Quintela‐Fandino Miguel

Radosa Julia

Rai Taranjit

Rajpert‐De Meyts Ewa

Ralph Stephen J

Ramaswamy Vijay

Rameshwar Pranela

Rao Chinthalapally V

Ray Partha

Reeh Matthias

Reifenberger Guido

Reuben James

Rezaie Ghashin

Rezsohazy René

Ricchetti Miria

Ricci‐Vitiani Lucia

Ricklefs Franz

Rincon Mercedes

Ringborg Ulrik

Rishi Arun K

Rix Uwe

Rizos Helen

Robak Pawel

Robbins Hilary

Roberti Maria Paula

Roberts Tara

Robertson Keith

Robinson Nirmal

Rodrigues‐Junior Dorival Mendes

Rodriguez‐Antona Cristina

Roesch Alexander

Roessler Stephanie

Roffe Martin

Rolny Charlotte

Romancer Muriel Le

Romero‐Córdoba Sandra

Rosell Rafael

Rosenfeld Michael Geoffrey

Rosselló‐Tortella Margalida

Rota Rosella

Rothwell Dominic Graham

Roukos Vassalis

Ruma I Made Winarsa

Russo Antonio

Sahai Erik

Sakamaki Jun‐ichi

Saloura Vassiliki

Santamaria David

Santarpia Libero

Santoni‐Rugiu Eric

Sayan Emre

Scherer Dominique

Scherer Florian

Schewe Denis M

Schneegans Svenja

Scholler Nathalie

Schueller Ulrich

Schulte Gunnar

Schuuring Ed

Schüz Joachim

Scutts Simon

Seano Giorgio

Seewaldt Victoria L

Senapati Dhirodatta

Serra Violeta

Servetto Alberto

Sevenich Lisa

Shachar Idit

Shai Yechiel

Shan Chao

Shao Chen

Shaul Yoav D

Shaw Andrey

Shen Han Ming

Shi Huidong

Shi Qihui

Shubeita George

Siddharth Sumit

Silke John

Silva Ligia

Simon SM

Simpson Andrew

Sinn Marianne

Sitcheran Raquel

Skånland Sigrid

Slade Neda

Smirnov Artem

Sodir Nicole

Son Ji Woong

Sonneveld Pieter

Soto Ubaldo

Spassieva Stefanka

Spindler Volker

Sproll Christoph

Squatritto Massimo

Srivastava Sanjay K

Staaf Johan

Staples Christopher

Stebbing J

Stein Ulrike S

Stern Peter

Stoecklein Nikolas H

Strati Areti

Su Lee Yeon

Sugimura Haruhiko

Sukocheva Olga

Sukumar Saraswati

Sun Cheng‐Cao

Sun Shisheng

Sun Xueying

Surralles Jordi

Suzuki Hiromu

Sztupinszki Zsofia

Szymanski de Toledo Marcelo

Taghizadeh Hossein

Tait Stephen

Tamminga Menno

Tang Chad

Tang Ranran

Tavakol Shima

Telleria Carlos M

ten_Dijke Peter

Teppan Julia

Terlecki‐Zaniewicz Stefan

Terstappen Leon WMM

Theillet Charles

Thomas Frederic

Tian Ye

Timpson Paul

Togawa Kayo

Toki Maria

Tom John

Tomar AK

Tomlinson Michael G

Tommasi Stefania

Tommasino Massimo

Tong Man

Toyoda Hidenori

Tressler Caitlin

Trisciuoglio Daniela

Triulzi Tiziana

Tullo Apollonia

Turashvili Gulisa

Uras Iris

Van den Eynden Jimmy

van der Leest Paul

van Diest Paul J

van Kempen Léon C

van Kruchten Michiel

Van Seuningen Isabelle

Van Tine Brian

van Vugt Marcel

Vandenberg Cassandra

Vaquero Javier

Varadarajan Sankar

Vasanthakumar Ajithkumar

Vaux David

Velazquez Miguel

Verginelli Federica

Vermeulen Louis

Vibhakar Rajeev

Vicent Silvestre

Vieira Andre

Vieito Maria

Vishwanatha Jamboor K

Vitale Ilio

Vivanco María del Mar

Vlashi Erina

Volinia Stefano

Volkmer Beate

von Amsberg Gunhild

von Bubnoff Nikolas

Voon Dominic Chih‐Cheng

Waghmare Sanjeev

Wallace Nicholas

Wang Chenji

Wang Kang

Wang Liming

Wang Lisheng

Wang Lizhong

Wang Tianzhen

Wang Wengong

Wang Xiongjun

Wang Zhirui

Watanabe Massayuki

Waterton John

Wehrle Julius

Weigelt Britta

Weisberg Ellen L

Wert Lamas Leon

Wesche Jørgen

Weyergang Anette

Wiemann Stefan

Wietrzyk Joanna

Wijnenga Maarten

Wikman Harriet

Wikström Pernilla

Wilking Nils

Williams Dr

Wiman Klas G

Windle Brad

Winkler Frank

Witz Isaac P

Wong Alan SL

Wozniak Agnieszka

Wright Stuart

Wu Bin

Wu Chao

Wu Sihan

Wyatt Alexander

Xiao Yichuan

Xie Ruiling

Xing Deyin

Xu Bin

Xu Bo

Xu Dawei

Xu Feiyue

Xu Naihan

XuEugenia Y

Yaeger R

Yang Bin

Yang Yungui

Yao Tso‐pang

Yao Yucheng

Yarden Yosef

Yelamos Jose

Yochum Gregory

Young Ken H

Yu Chong‐Jen

Yu Riyue

Zamarchi Rita

Zawacka‐Pankau Joanna

Zeeshan Saman

Zeng Xiaowei

Zhan Cheng

Zhang Dongya

Zhang Fan

Zhang Honghe

Zhang L

Zhang Lei

Zhang Peipei

Zhang Yilei

Zhang Zhen

Zheng Mingjun

Zhivotovsky Boris

Zhou Hu

Zhou Meng

Zhu Gui‐qi

Zhu Lixin

Zhu Wencheng

Zhu Xiao

Zhuo Wei

Zimmer Yitzhak

Zitzelsberger Horst

Zoni Eugenio

Zugazagoitia Jon

